# An automated method for analysis of microcirculation videos for accurate assessment of tissue perfusion

**DOI:** 10.1186/1471-2342-12-37

**Published:** 2012-12-21

**Authors:** Sumeyra U Demir, Roya Hakimzadeh, Rosalyn Hobson Hargraves, Kevin R Ward, Eric V Myer, Kayvan Najarian

**Affiliations:** 1 Signal Processing Technologies LLC, Richmond, VA, USA; 2 Department of Electrical Engineering, Virginia Commonwealth University, Richmond, VA, USA; 3 Department of Emergency Medicine, Virginia Commonwealth University, Richmond, VA, USA; 4 Virginia Commonwealth University Reanimation Engineering Science (VCURES), Richmond, VA, USA; 5 Department of Computer Science, Virginia Commonwealth University, Richmond, VA, USA; 6 Department of Emergency Medicine, Michigan Center for Integrative Research in Critical Care, University of Michigan, Ann Arbor, MI, USA

## Abstract

**Background:**

Imaging of the human microcirculation in real-time has the potential to detect injuries and illnesses that disturb the microcirculation at earlier stages and may improve the efficacy of resuscitation. Despite advanced imaging techniques to monitor the microcirculation, there are currently no tools for the near real-time analysis of the videos produced by these imaging systems. An automated system tool that can extract microvasculature information and monitor changes in tissue perfusion quantitatively might be invaluable as a diagnostic and therapeutic endpoint for resuscitation.

**Methods:**

The experimental algorithm automatically extracts microvascular network and quantitatively measures changes in the microcirculation. There are two main parts in the algorithm: video processing and vessel segmentation. Microcirculatory videos are first stabilized in a video processing step to remove motion artifacts. In the vessel segmentation process, the microvascular network is extracted using multiple level thresholding and pixel verification techniques. Threshold levels are selected using histogram information of a set of training video recordings. Pixel-by-pixel differences are calculated throughout the frames to identify active blood vessels and capillaries with flow.

**Results:**

Sublingual microcirculatory videos are recorded from anesthetized swine at baseline and during hemorrhage using a hand-held Side-stream Dark Field (SDF) imaging device to track changes in the microvasculature during hemorrhage. Automatically segmented vessels in the recordings are analyzed visually and the functional capillary density (FCD) values calculated by the algorithm are compared for both health baseline and hemorrhagic conditions. These results were compared to independently made FCD measurements using a well-known semi-automated method. Results of the fully automated algorithm demonstrated a significant decrease of FCD values. Similar, but more variable FCD values were calculated using a commercially available software program requiring manual editing.

**Conclusions:**

An entirely automated system for analyzing microcirculation videos to reduce human interaction and computation time is developed. The algorithm successfully stabilizes video recordings, segments blood vessels, identifies vessels without flow and calculates FCD in a fully automated process. The automated process provides an equal or better separation between healthy and hemorrhagic FCD values compared to currently available semi-automatic techniques. The proposed method shows promise for the quantitative measurement of changes occurring in microcirculation during injury.

## Background

Understanding the distribution and circulation of blood in capillaries has been considered a key aspect for assessment of tissue perfusion [1]. Visualization and quantification of changes in microcirculation has been proposed as a potential tool in diagnosis and treatment of illnesses and diseases such as sepsis [2], sickle cell disease [3,4], chronic ulcers, diabetes mellitus and hypertension [5,6]. In each of these diseases, several characteristics of the microcirculation such as the structure of capillaries and quality of blood flow in the capillaries change over time [7-11]. A recent study suggests there is value in monitoring the microcirculation for titrating vasodilators in perioperative use [12]. Monitoring microcirculation during resuscitation could also be envisioned as a tool to prevent over and under resuscitation of victims of hemorrhage and other critical illness and injuries such as sepsis. Therefore, quantitative and accurate analysis of the microcirculation is likely to be essential if microcirculatory imaging is to be adopted as useful tool in clinical monitoring [13].

Orthogonal polarization spectral (OPS) imaging [1,2,5] and side-stream dark field (SDF) imaging [13,14] have been extensively employed in the field of clinical microcirculatory research. OPS and SDF imaging are both non-invasive imaging modalities and have been used to track changes in the microcirculation on mucosal surfaces. Most studies have used the sublingual surfaces of the oral cavity. These imaging techniques use green polarized light with wavelength of 550 nm which is absorbed by hemoglobin and makes red blood cells visible [15,16].

To analyze microcirculatory images and videos, several software tools have been developed. However currently available software is unable to perform real or near real time analysis of the videos and require manual intervention to ensure accurate results. Researchers in the field have stated the need for improvements of current software to expedite clinical bedside use [17]. The computer-assisted image analysis system CapImage (Zeintl, Heidelberg, Germany) was originally developed for traditional intravital microscopy [18], but is capable of analysis of SDF and OPS images [19]. It uses a Line Shift Diagram Method for measurement of velocity and real-time movement correlation. This software tool is capable of measuring different properties of the microcirculation such as blood cell velocity and capillary density. However, it is only capable of detecting straight blood vessels which limits its efficacy since the microvascular geometry is complex. Expert users of CapImage claim that analysis of microcirculation with CapImage is time consuming and may only be performed off-line [20].

CapiScope, a system for the measurement of capillary morphology and capillary blood cell velocity, requires stable images, but lacks a stabilization function [21]. JavaCap and Capilap Toolbox are two other available software tools which use triangulation methods to calculate intercapillary distance [22,23].

Automated Vascular Analysis (AVA)—also known as MAS (Microvascular Analysis Software, Microvision Medical BV)—is the most current commercial software tool developed by Dobbe et al [24] for analysis of microcirculation videos. The method is the most accurate among the existing systems and performs a semi-automated process based on image stabilization, centerline detection and space time diagram. Despite all the capabilities provided by AVA, it does not provide full automation which leaves the burden of selecting the areas of interest, configuration, initialization, filtering of many false positives, dealing with many false negatives, and addressing of connectivity to the user. In addition, according to the developers of AVA, the software provides neither automatic vessel detection nor vessel diameter and blood flow calculation [24]. The system, while an improvement, requires manual editing which can take over 20 minutes for a typical video sequence.

The aim of this study is to automate the analysis of microcirculatory video recordings and the derivation of Functional Capillary Density (FCD). FCD is defined as the ratio of the area of functionally active capillaries to the entire area of the image. FCD has been considered an important measurement of the microcirculation to indicate the quality of tissue perfusion [25]. Image processing algorithms are designed for this study to automatically detect capillaries and small blood vessels in order to derive diagnostically useful information that may assist clinicians and medical researchers in the future.

## Methods

**Figure 1:**
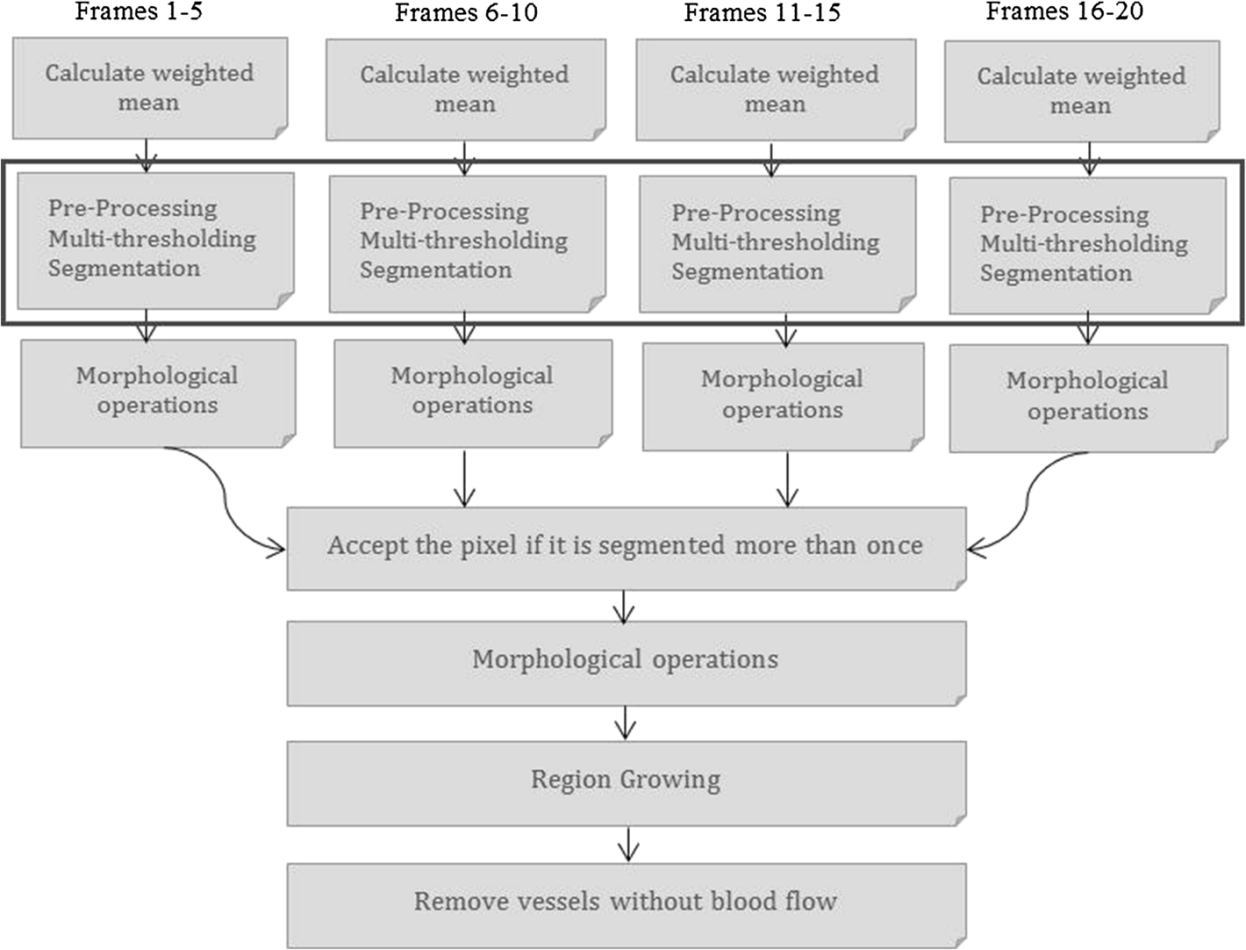
**Schematic of the proposed methodology.** Proposed methodology is summarized in Figure 1. Stabilization of the videos is not included in the schematics. After stabilization, the weighted mean of five consecutive frames is calculated and the preprocessing and segmentation algorithms are applied on the mean frame. Averaging more than five frames results in “over-averaging” phenomena that would eliminate some of the important features important for segmentation. Experimentally, our results indicate that the 5-frame approach provides the best results. After the segmentation, the segmented frames are combined together to generate one single binary image and calculate Functional Capillary Density from this binary image.

**Figure 2:**
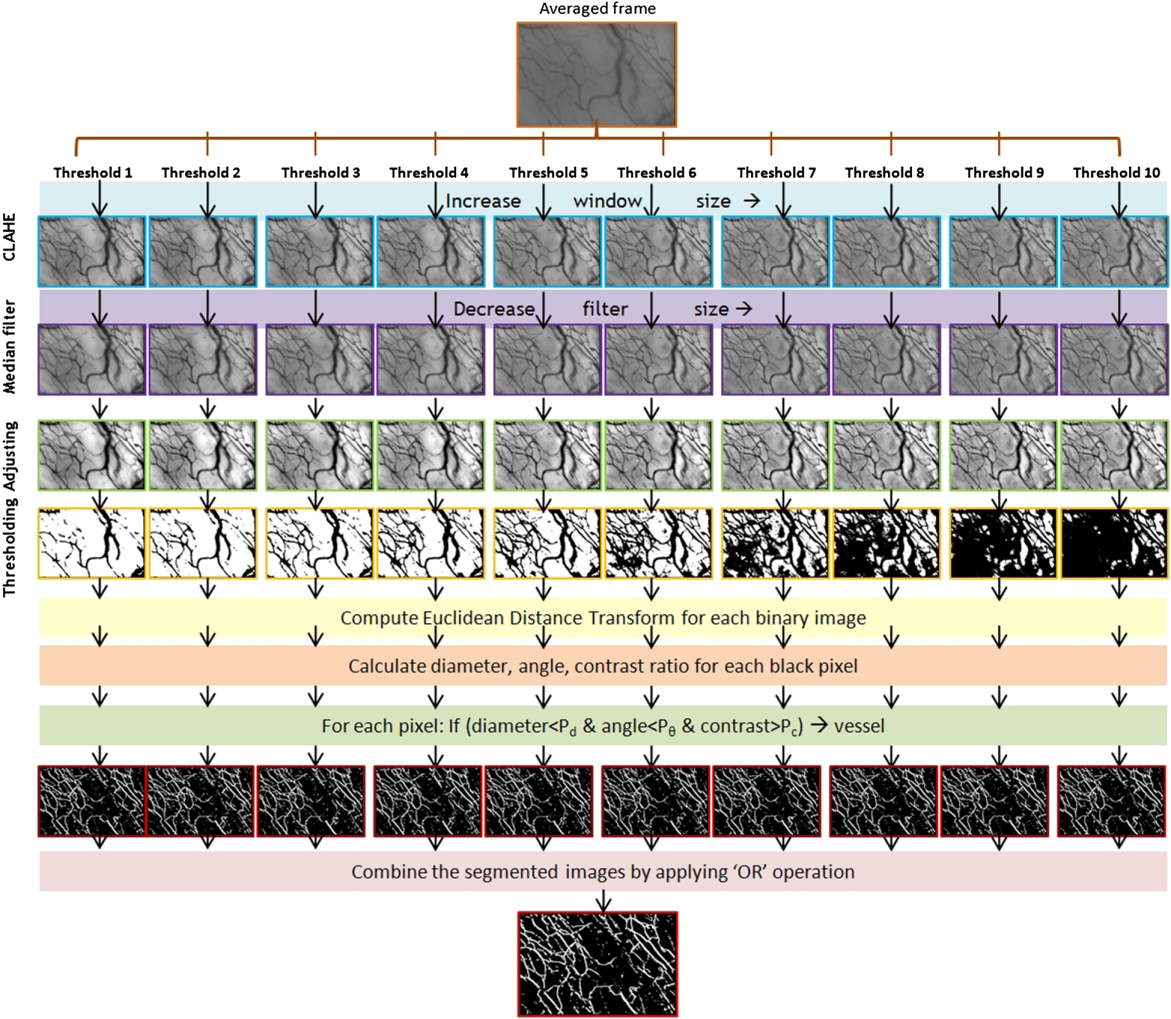
**Detailed diagram of pre-processing and vessel segmentation.** Figure 2 provides a detailed diagram of preprocessing and segmentation steps for different threshold levels. It starts with the averaged frame. For 10 different threshold levels, the parameters of CLAHE (Contrast Limited Adaptive Histogram Equalization) and median filter vary throughout the process. For the first threshold level, the window size of CLAHE is kept small. Median filter is applied right after histogram equalization with a small filter size such as 3 × 3. Median filtering is followed by image adjustment. The preprocessed image is converted to binary image using the first threshold level. Euclidean Distance Transform (EDT) is calculated for the binary image. Diameter and angle parameters are extracted from EDT and with the addition of contrast ratio; three parameters are used to determine if a pixel belongs to a vessel.

The methodology behind the proposed algorithm to quantify the assessment of the microcirculation is summarized in Figure 1. The process—which is not shown in the schematic diagram—starts with the stabilization of the video frames. The weighted mean of consecutive frames from the stabilized video is calculated for each five-frame block. Pre-processing, multi-thresholding and vessel segmentation are the following steps and are highlighted in the diagram. A more detailed diagram is provided in Figure 2 to describe pre-processing, multi-thresholding and segmentation algorithm. After performing morphological operations such as filling and opening, the binary images resulting from segmentation are unioned together. If a pixel is segmented as vessel in more than one frame, it is assigned the label of “vessel”. Post processing includes additional morphological operations (e.g., bridge, spur, fill) and region growing to eliminate any possible discontinuities. Vessels with blood flow—perfused vessels—are identified in the last step and FCD is calculated accordingly.

### Stabilization

The microcirculatory videos are captured by a commercially available hand-held SDF device (Microscan, Microvision Medical, BV). Because it is hand-held and is highly susceptible to motion artifacts due to movement of subject and/or device, we developed a stabilization algorithm to eliminate these motion artifacts. A block matching algorithm is developed that calculates cross-correlation coefficients to measure the similarity of the blocks. Block matching algorithms use a predefined size of windows-blocks or even entire images to estimate motion vectors. One of the disadvantages of block matching methods is defined as 'remarkableness' of the window content [26]. If a window does not contain distinctive details, there is a high probability of mismatch. To avoid errors caused by ‘remarkableness’ issues, i.e. using points for matching that have no significant image-processing values (e.g. regular pixels inside the background) the processed blocks are checked to ensure they include blood vessels using Laplacian of Gaussian filtering. The stabilization process is described in detail in a previously published study [27]. Gradients of the frames are calculated using the first order derivative of the Gaussian function. Gradient of the Gaussian enhances images and improves visibility of blood vessels. Distinctive features of the images, which are typically the branching points of blood vessels, are selected and assigned as control points to overcome remarkableness issues. Control points are selected which are known to belong to capillaries to calculate the transformation between two consecutive frames. For this purpose, a 3-by-3 Laplacian filter which calculates second order derivatives is applied to the output of the Gaussian Gradient.

The maximum values from seven areas of the frame are selected as control points. Then, around the control points, 25 × 25 pixel windows are selected as sub-regions. The cross-correlation is calculated between these sub-regions in the current frame and a 40 × 40 pixel window around these sub-regions in the following frame. The size of the windows discussed above was optimized based on a previous visual empirical assessment of the algorithm over a set of microcirculation videos. The frames are registered according to the results of correlation calculations. Since the control points are defined for the first frame and they are tracked through adjacent frames, Laplacian filtering is not repeated throughout the algorithm. If any of the defined control points leaves the current frame due to excessive motion, new control points are defined using the same method.

### Preprocessing, multi-thresholding, and segmentation

After the stabilization of the video recordings, the weighted mean of five consecutive frames is calculated to improve connectivity of the blood vessels. The frame in the middle has the highest weight in this process. Averaging the frames is followed by preprocessing algorithms. The vessel extraction algorithm is based on multiple level thresholding. Preprocessing is repeated at each threshold with different parameters.

Video contrast and clarity vary widely from source to source and necessitate preprocessing in order to generate images that will yield accurate results. To enhance the local contrast, Contrast Limited Adaptive Histogram Equalization (CLAHE) is performed on microcirculation images. CLAHE partitions the image into small regions, called ‘tiles’, and applies histogram equalization to each tile in order to even out the overall gray level distribution of the image [28]. Histogram equalization is followed by median filtering. To remove noise, median filtering is a widely preferred method in the literature [29]. In this application, the purpose of applying median filtering is smoothing.

The window sizes of adaptive histogram equalization and median filtering are subject to change at each threshold level. At low threshold levels, in order to include only wide and clear vessels, histogram equalization is applied in smaller windows. The median filter size is kept large at these low threshold levels. As the threshold level increases, the result of the process is a darker binary image with almost all vessels and background included. Window size of adaptive histogram equalization is increased and median filter size is reduced to enhance the thinner vessels. Without these steps results will suffer as vessels may not be fully segmented and flow correctly detected. This preprocessing is followed by thresholding.

Vessel segmentation is based on verifying each pixel at multiple threshold levels as vessel. The method is modified from a pixel verification method proposed by Jiang et al. using retinal images [30]. The pre-processed images are converted to binary images using multiple threshold levels resulting in multiple binary images. Euclidean Distance Transform (EDT) is created for each binary image. EDT calculates the distance of nearest background pixel for each object pixel. The coordinates of the nearest background pixel is the second output of the transform. For each pixel of each binary image, three different features are calculated using the outputs of the EDT and the gray-level intensity values of the pixels. Two of these parameters are the diameter *P*_*d*_ and the angle *P*_*θ*_ of the vessel, which are considered as geometrical features. The gray level intensity values of pre-processed images are used to calculate the third feature, *P*_*C*_, which is the contrast ratio identifying the ratio of intensity across background and blood vessel pixels. These three features serve to determine if a pixel in the image is indeed a vessel pixel. Pd limits the size of the vessel to ensure it is a capillary. *P*_*θ*_ ensures curvilinear structure to the pixel, and excludes any anomalous pixels because of physiological improbabilities. Pc, ensures contrast between background and vessel pixels.

**Figure 3:**
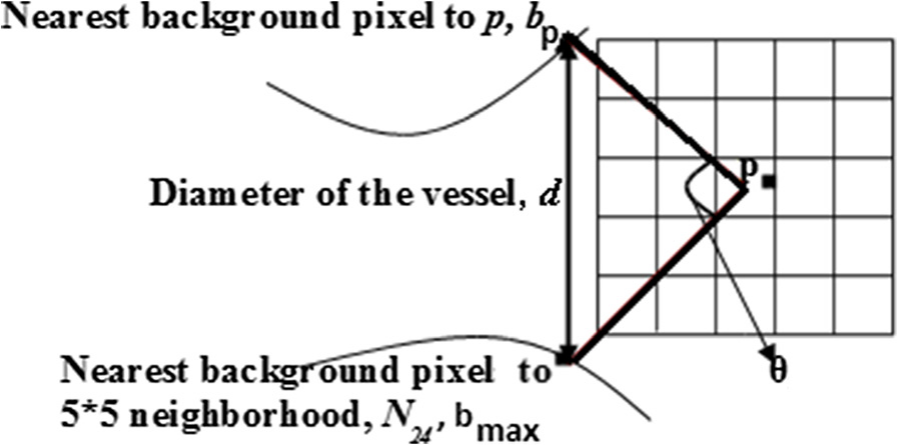
**Method of validating vessel pixels.** A vessel candidate pixel is labeled as *p* in Figure 3. The output of EDT is used to find the nearest background pixel to *p*, *b*_*p*_. For each of the 24 neighboring pixels in the 5 × 5 neighborhood around *p*, *n*_1_ − *n*_24_, the nearest background pixel is found, *b*_*n*_, and used to calculate the diameter, angle and contrast ratio values. The *b*_*n1-24*_ having the greatest distance from *b*_*p*_ is considered the opposite background pixel, *b*_*max*_, and the distance is the diameter, *d*, of the vessel. If *d* is less than *P*_*d*_ then the angle, θ, is calculated between *b*_*p*_, *p* and *b*_*max*_ and is used to validate the distance by ensuring that *b*_*p*_ and *b*_*max*_ are on opposite sides of the vessel (θ must be greater than *P*_*θ*_). Finally, the contrast ratio between *p* and *b*_*max*_ is calculated and if greater than *P*_*c*_, the candidate pixel is considered a valid vessel pixel. Since the found vessels lie along the center of the actual vessel, the vessel must be reconstructed using the found diameter and pixel locations.

Figure 3 provides a visual depiction of these parameters. 5*5 neighborhood of a candidate pixel is included in the calculations. The current pixel is referred to as ‘*p*’, which is a vessel candidate. Its 24 neighbors are defined as *N*_1_ − *N*_24_. The nearest background pixel to ‘*p*’ is ‘*b*_*p*_’ and the nearest background pixels to each of the 24 neighbors are ‘*b*_*n*1_ − *b*_*n*24_’. The *b*_*n1-24*_ having the greatest distance from *b*_*p*_ is considered the opposite background pixel, *b*_*max*_. The maximum Euclidean distance from ‘*b*_*p*_’ to ‘*b*_*n*1_-*b*_*n*24_’ is decided to be the diameter of the candidate pixel:

(1) $$d=\max_{\text{bj}\in b_{n1}-b_{n24}}\left\|\bar{b_{p},b_{j}}\right\|$$

where $\left\|\bar{b_{p},b_{j}}\right\|$ is the distance between *b*_*p*_ and *b*_*j*_. The parameter θ is the angle between the background pixels ‘*b*_*p*_’ and ‘*b*_*n*1_-*b*_*n*24_’ according to Figure 3. The angle is calculated using the cosine rule. If *d* is less than *P*_*d*_ then the angle, θ, is calculated between *b*_*p*_, *p* and *b*_*max*_. The maximum angle derived from the 24 neighbor pixels is used as ‘θ’:

(2) $$\theta =\max_{\text{bj}\in b_{n1}-b_{n24}}\cos^{-1}\left(\frac{{\left\|\bar{p,b_{p}}\right\|}^{2}+{\left\|\bar{p,b_{j}}\right\|}^{2}-d^{2}}{2\left\|\bar{p,b_{p}}\right\|\left\|\bar{p,b_{j}}\right\|}\right)$$

Finally the third feature is the ratio of gray level values of nearest background pixels and the vessel candidate ’*p*’:

(3) $$C=\max_{\text{bj}\in b_{n1}-b_{n24}}\frac{\text{GL}\left(b_{j}\right)}{\text{GL}\left(p\right)}$$

where *GL(p)* is the gray-level intensity value of current pixel ’*p*’. To calculate contrast ratio, images with enhanced contrast are used. Therefore, *GL* in Equation 3 stands for the gray level of the output of the CLAHE.

To verify the vessel candidate pixel, ’*d*’ needs to be less than pre-defined *P*_*d*_ to avoid large vessels, ’θ’ needs to be larger than *P*_*θ*_ to assure curvilinear structure and the calculated contrast ratio needs to be higher than *P*_*C*_ to remove background noise. If the pixel in the binary image is black and it meets the criteria defined by three parameters *P*_*d*_, *P*_*θ*_ and *P*_*C*_, it is verified to be a vessel pixel. After repeating the same procedure for all threshold levels, the segmented images of each threshold level are combined resulting in one segmented binary image for each frame.

The parameters, *P*_*d*_, *P*_*θ*_ and *P*_*C*_ were selected from multiple experiments using a set of training videos. *P*_*d*_ controls the maximum diameter of the blood vessels to accept. It is determined based on the diameter of blood vessels and capillaries to be included in the microcirculation. *P*_*θ*_ is another geometric parameter to ensure curvilinear structure of the vessels and is empirically derived.

### Region growing

Since the segmentation algorithm is based on pixel verification, it is possible to have isolated pixels in the result. To prevent that, binary morphological operators are used before region growing. The morphological operators include filling the isolated interior pixels and opening.

The region growing algorithm is developed to overcome disconnectivity of blood vessels. First, the final segmented image is divided into 35∗35 windows to determine the orientation of segmented vessel within the window. The vessel is allowed to grow in the computed direction if the gray level is within the range of average gray level of vessel pixels in the window ±0.5∗*standard deviation*.

### FCD calculation

Segmentation processes described up until this stage detect all blood vessels at each frame of the video recording. To provide quantitative information on blood flow, the vessels through which blood is flowing must be identified. To that end, the difference of consecutive segmented frames is calculated pixel by pixel. If the summation of difference for twenty segmented frames is higher than a threshold value, the pixel is assigned as an active blood vessel.

FCD is currently one of the main parameters used to evaluate the microcirculation. FCD can be calculated using two different approaches: one is completely manual by gridding the frame and counting the number of vessels crossing the grid lines; the second approach calculates the ratio of perfused vessels to the total surface using a software tool [31]. FCD is calculated automatically in this study by dividing the area of active vessels to the total area of interest. It is much easier to form the skeleton of the network of active capillaries and calculate the length of this skeleton to form the density measure. However, since the width/thickness of capillaries along this network would be inconsistent (on the actual sublingual surface, the captured video, and in the processed image), the density measure calculated on this length would be the least reliable measure, as it does not incorporate the changes in the thickness of the capillary and therefore the true extent of circulation inside the capillary. The area-based density measure, on the other hand, since it incorporates the thickness of the capillaries into the calculation, is not susceptible to this issue. We have also included the length-based FCD calculation in this paper for comparison with the output from AVA.

## Results and discussion

### Results

**Figure 4:**
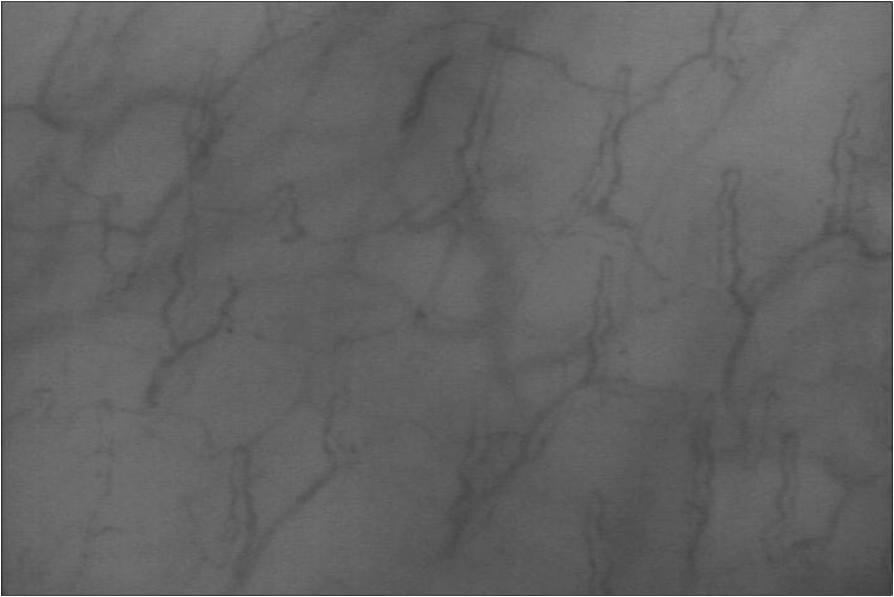
**An example frame from a healthy subject.** An example frame from a sublingual microcirculatory video captured from a healthy baseline subject is presented.

**Figure 5:**
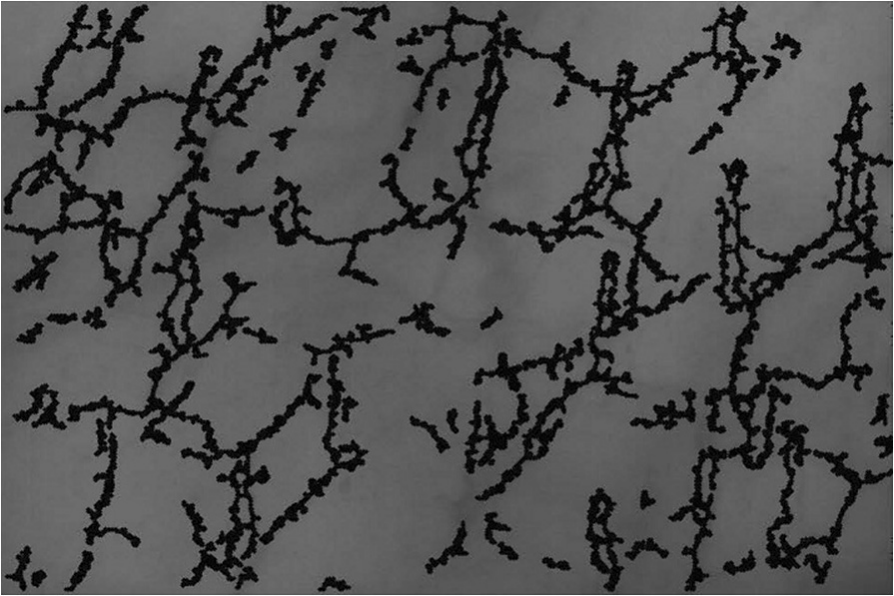
**Segmented active capillaries of the frame in Figure**4**.** The result of the proposed algorithm highlights all active capillaries from the original frame in Figure 4.

The proposed experimental algorithm and the software product Microcirculation Analyzer (MCA) were applied to videos acquired from a library of microcirculatory videos of a previous animal study. The protocol was approved by the Virginia Commonwealth University Institutional Animal Care and Use Committee in accordance with the National Institutes of Health Guide for the Care and Use of Laboratory Animals (National Institutes of Health Publication 86-23, revised 1996). In this animal study, sublingual microcirculatory videos were taken from nine healthy juvenile swine at baseline as well as after 40% of the animal’s blood was removed. All animals were under a state of general anesthesia. Twenty frames from each video were used for the assessment of the video recordings. The parameters at multi-thresholding stage are defined empirically. Specifically, a series of videos were used as the training set in which we change the values of these parameters over a reasonable range and choose the values that give the parameters providing the best segmentation results. In this study, “the best segmentation result” was visually evaluated. According to this process the image features are obtained; *P*_*d*_ =13, *P*_*θ*_ =130 and *P*_*C*_ = 1.17. The angle is calculated in degrees. In order to capture desired vessels and avoid segmenting larger structures like venules, *P*_*d*_ =13 corresponds to a vessel diameter of about 20 μm at the resolution captured by the test camera. An original frame from sublingual microcirculatory video of a healthy subject is displayed in Figure 4. Active capillaries segmented using MCA are highlighted in Figure 5.

FCD parameters are calculated from SDF videos for nine subjects in both baseline (PPV = 1) and hemorrhage (PPV < 1) conditions. As expected, a significant decrease in FCD is noticed during hemorrhage recordings with respect to baseline videos. For MCA, a paired-samples t-test was conducted to compare FCD values of healthy baseline and hemorrhage video recordings. There was a significant difference in the scores for healthy baseline (*μ* = 12.68, *σ* = 1.479, area-based; *μ* = 3.26 × 10^-5^, *σ* = 5.93 × 10^-6^, length-based) and hemorrhage (*μ* = 7.35, *σ* = 2.139, area-based; *μ* = 1.99 × 10^-5^, *σ* = 5.53 × 10^-6^, length-based) conditions; with *t*_*8*_ = 6.50 and *p-value* = .000189. These results suggest that the proposed algorithm, MCA, can successfully derive quantitative information from microcirculation videos. Specifically, the results suggest that microcirculatory alterations caused by hemorrhage can be identified by analyzing sublingual microcirculatory video recordings. FCD percentages for nine subjects for healthy baseline and hemorrhage conditions are shown in Table 1.

**Table 1:** Calculated FCD values using the automated MCA algorithm, including both area and length based results

	**Baseline (PPV = 1)**		**Hemorrhagic (PPV < 1)**		**Difference**	
	**FCD (Area) %**	**FCD (Length) mm/mm**^**2**^	**FCD (Area) %**	**FCD (Length) mm/mm**^**2**^	**FCD (Area) %**	**FCD (Length) mm/mm**^**2**^
Subject 1	12.16	2.35 × 10^-5^	9.72	1.42 × 10^-5^	2.44	9.28 × 10^-6^
Subject 2	14.99	3.35 × 10^-5^	7.32	1.48 × 10^-5^	7.67	1.87 × 10^-5^
Subject 3	13.97	3.10 × 10^-5^	11.38	3.26 × 10^-5^	2.59	-1.54 × 10^-6^
Subject 4	11.95	2.56 × 10^-5^	5.92	1.97 × 10^-5^	6.03	5.91 × 10^-6^
Subject 5	10.21	3.10 × 10^-5^	7.58	2.02 × 10^-5^	2.63	1.08 × 10^-5^
Subject 6	13.81	3.75 × 10^-5^	4.41	2.12 × 10^-5^	9.40	1.63 × 10^-5^
Subject 7	11.90	4.01 × 10^-5^	7.38	1.65 × 10^-5^	4.52	2.36 × 10^-5^
Subject 8	13.49	4.04 × 10^-5^	7.05	1.77 × 10^-5^	6.44	2.28 × 10^-5^
Subject 9	11.65	3.11 × 10^-5^	5.37	2.26 × 10^-5^	6.28	8.52 × 10^-6^
Mean	12.68	3.26 × 10^-5^	7.35	1.99 × 10^-5^	5.33	1.27 × 10^-5^

**Figure 6:**
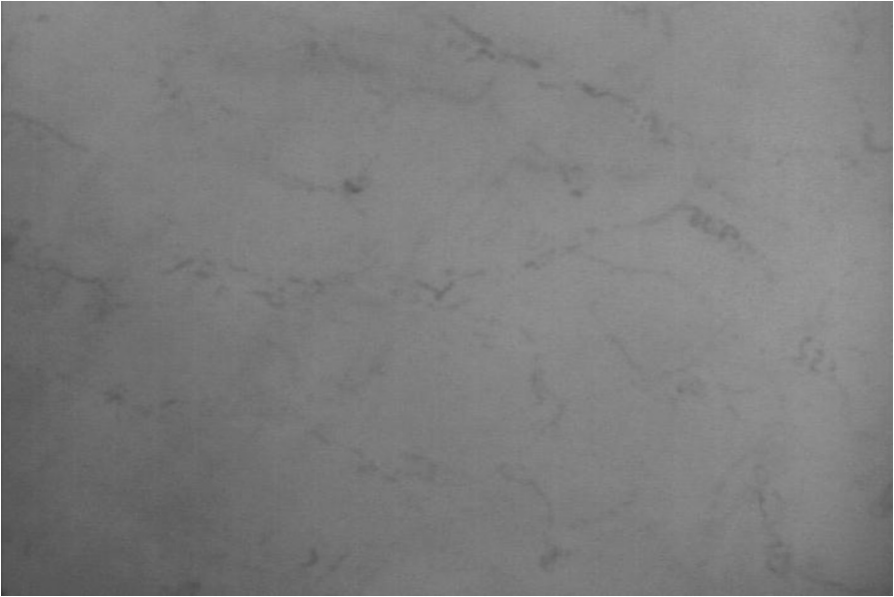
**Example of hemorrhage subject video source.** An original frame from sublingual microcirculatory video of a hemorrhage subject.

The same videos were analyzed using the currently available semi-automated tool, AVA, with manual editing. Analysis was performed using the method described in the manufacture’s tutorial of the product. All videos were analyzed in an identical format. The microcirculation videos are first automatically analyzed using the software tools and then manual interaction with the software for manipulation of the segmentation results must be performed to remove the large vessels, false positives, and vessels without flow. Figure 6 provides and example of the microcirculation after hemorrhage and can be compared to the healthy (baseline) microcirculation in Figure 4.

Videos were analyzed by two experts previously trained with AVA and the results for each video averaged in the analysis of FCD. These individuals were blinded to FCD values derived from the fully automated method. The analysis resulted in a Kappa coefficient of 0.9 (very good agreement). The Kappa coefficient is a statistical measure of inter-rater agreement. The FCD results of this analysis are provided in Table 2. A significant difference is also noticed in these scores for healthy baseline conditions (μ = 12.26, σ = 1.759, area-based; *μ* = 1.65 × 10^-5^, *σ* = 5.485 × 10^-6^, length-based) versus hemorrhage conditions (μ = 8.56, σ = 1.432, area-based; *μ* = 1.20 × 10^-5^, *σ* = 4.124 × 10^-6^, length-based); with *t*_*8*_ = 4.19 and *p-value* = 0.003.

**Table 2:** Calculated FCD values using semi-automated software, AVA

	**Baseline (PPV = 1)**		**Hemorrhagic (PPV < 1)**		**Difference**	
	**FCD (Area) %**	**FCD (Length) mm/mm**^**2**^	**FCD (Area) %**	**FCD (Length) mm/mm**^**2**^	**FCD (Area) %**	**FCD (Length) mm/mm**^**2**^
Subject 1	13.77	1.63 × 10^-5^	9.72	9.52 × 10^-6^	4.05	6.73 × 10^-6^
Subject 2	14.50	2.15 × 10^-5^	7.32	1.47 × 10^-5^	7.18	6.80 × 10^-6^
Subject 3	10.81	1.72 × 10^-5^	11.39	1.62 × 10^-5^	-0.58	9.75 × 10^-7^
Subject 4	11.95	2.41 × 10^-5^	6.68	1.91 × 10^-5^	5.27	5.00 × 10^-6^
Subject 5	9.44	2.18 × 10^-5^	9.21	9.65 × 10^-6^	0.23	1.22 × 10^-5^
Subject 6	13.38	1.00 × 10^-5^	7.47	8.29 × 10^-6^	5.91	1.72 × 10^-6^
Subject 7	10.15	1.75 × 10^-5^	8.36	1.43 × 10^-5^	1.79	3.15 × 10^-6^
Subject 8	13.23	1.23 × 10^-5^	8.06	8.22 × 10^-6^	5.17	4.08 × 10^-6^
Subject 9	13.07	8.18 × 10^-6^	8.82	8.04 × 10^-6^	4.25	1.35 × 10^-7^
Mean	12.26	1.65 × 10^-5^	8.56	1.20 × 10^-5^	3.70	4.53 × 10^-6^

It should be pointed out that even though AVA allows manual interaction, the version we had access to does not allow the user to draw the capillaries to be included. If the user sees that a capillary has not been identified, the user must define an area that the vessel resides in and then have the program outline the vessel. However, in some instances, the program will still not outline the vessel for inclusion in the FCD calculations. The user cannot manually force the outlining of vessels.

**Figure 7:**
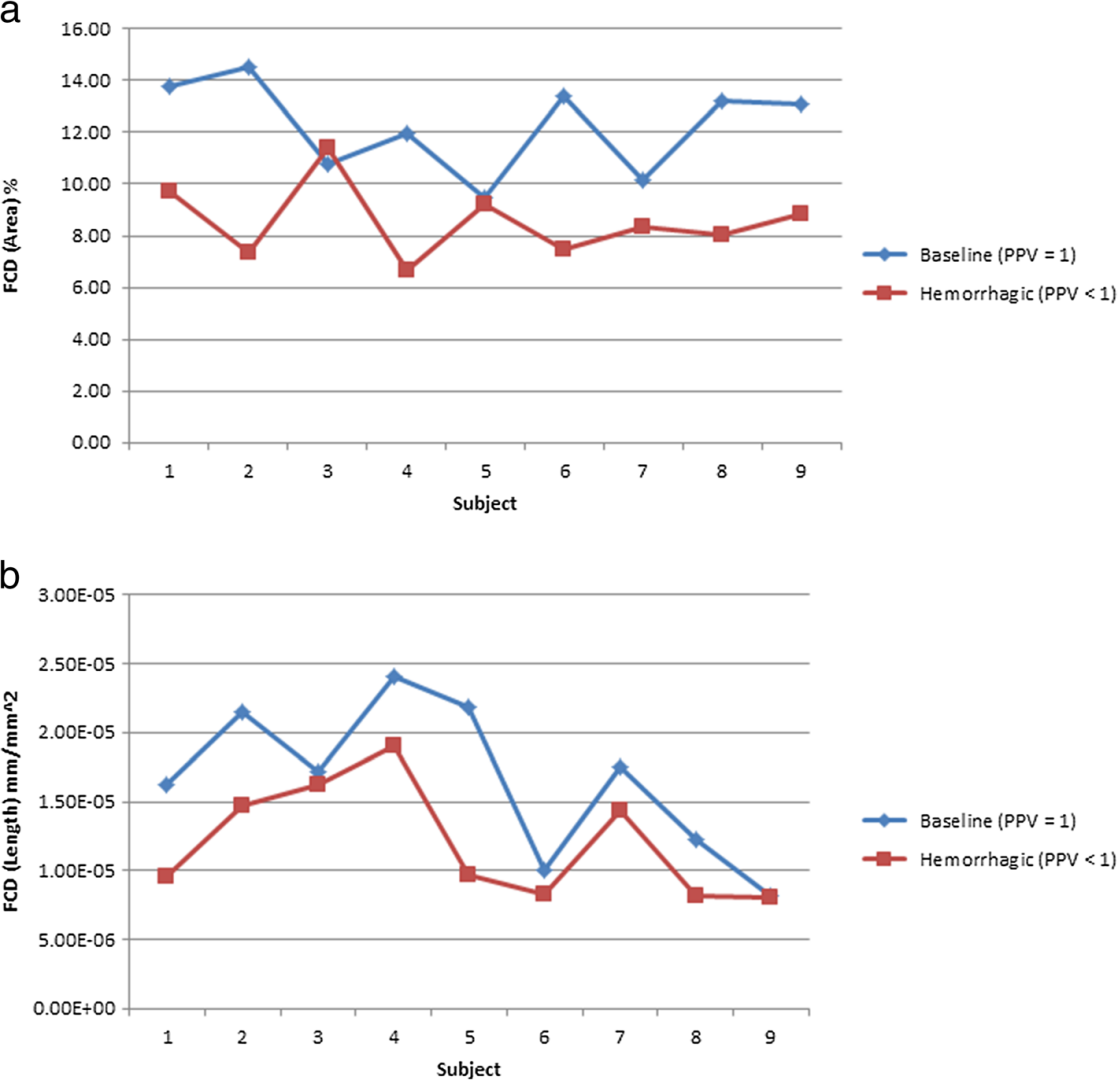
**FCD results calculated from heavily edited AVA.** The FCD values calculated from heavily edited AVA for both healthy baseline and hemorrhage conditions are displayed. The healthy condition FCD values are labeled as baseline. The change in FCD values during hemorrhage is not consistent. **a**: FCD results (area based) from heavily edited AVA show inconsistent separation between the healthy (baseline, PPV = 1) and hemorrhagic (PPV < 1) cases. **b**: FCD results (length based) from heavily edited AVA.

**Figure 8:**
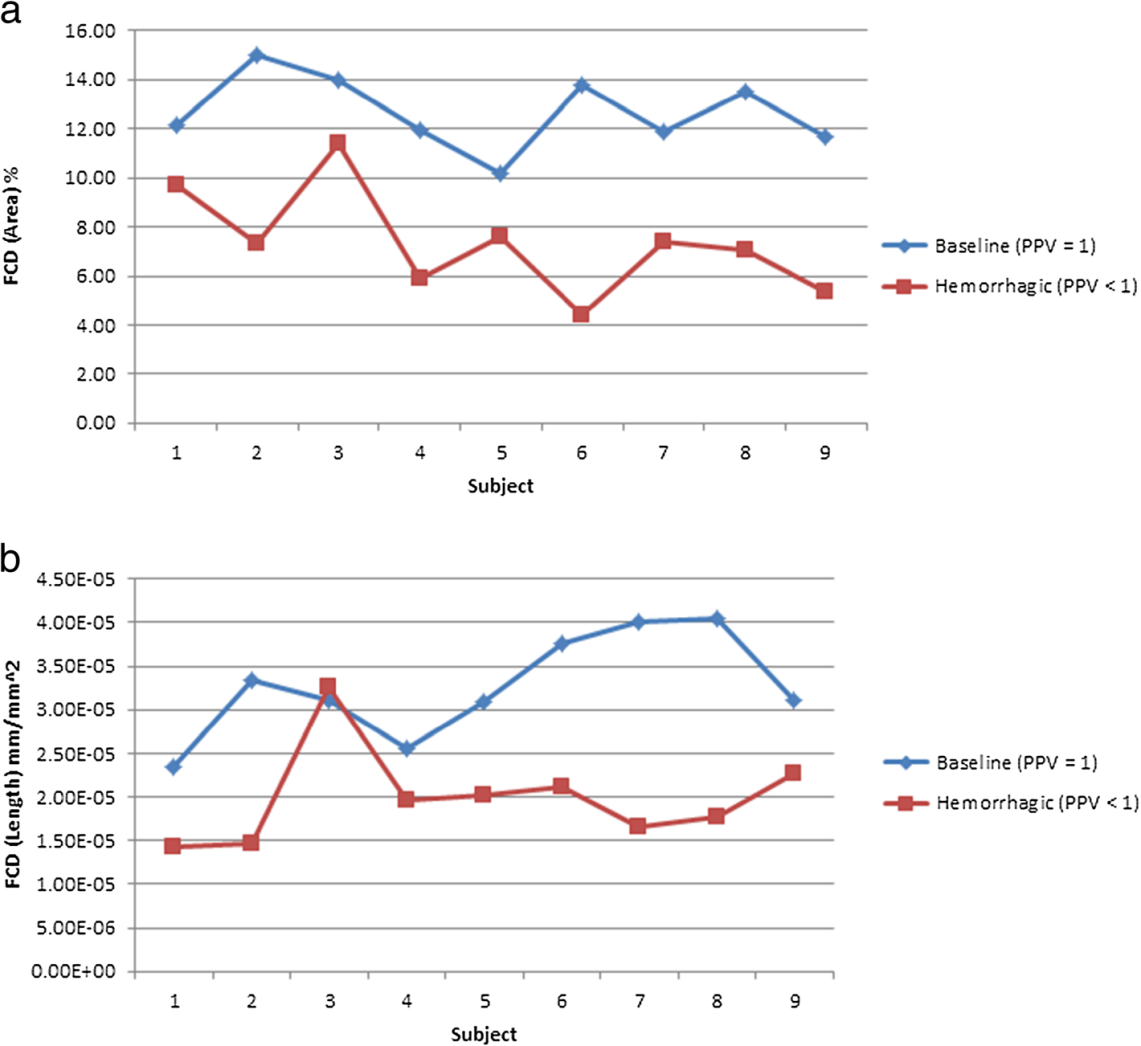
**FCD results calculated using the proposed algorithm (MCA).** The FCD values calculated using the proposed algorithm for both healthy baseline and hemorrhage conditions are displayed. The healthy condition FCD values are labeled as baseline. The decrease in FCD values for each subject during hemorrhagic is obvious in the provided figure. **a**: FCD results (area based) from the proposed automated system show better and consistent separation between healthy (baseline, PPV = 1) and hemorrhagic (PPV < 1) cases. **b**: FCD results (length based) from the proposed automated system show good separation, but demonstrate the problem with length based FCD calculation where vessel width is not taken into consideration as it is with area based FCD.

**Figure 9:**
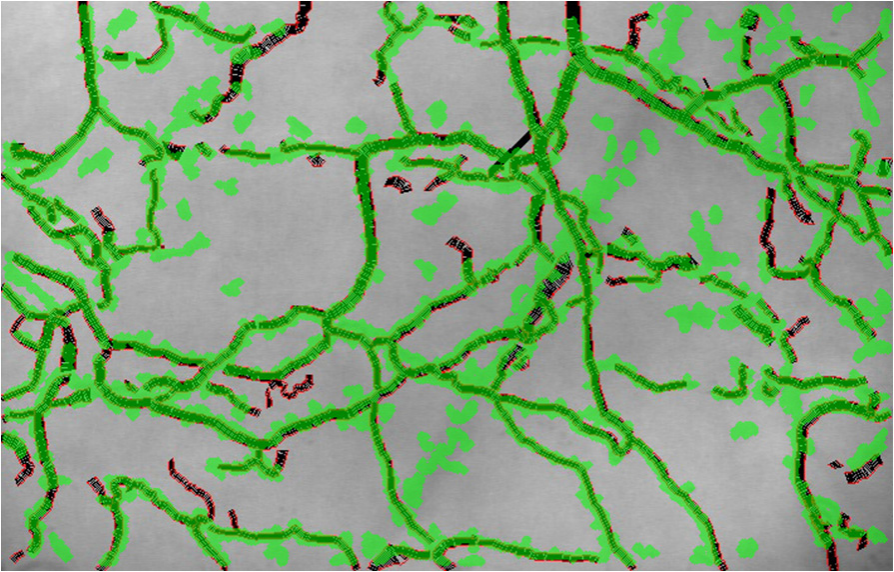
**Overlay of proposed automated method onto heavily edited AVA results showing a high degree of similarity.** Results from proposed automated method (green) superimposed over results from heavily edited AVA (red/black). Proposed method returns results 60 to 120 times faster than manual editing in AVA (20 seconds vs. 20-40 minutes).

**Figure 10:**
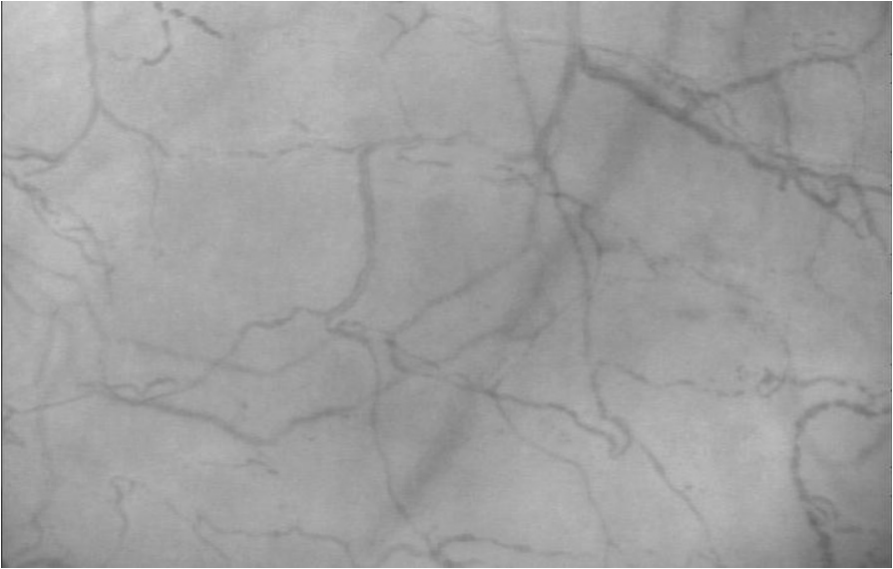
**Frame of video used for analysis in Figure**9**.** An example frame of the video used to generate Figure 9 in both MCA and heavily edited AVA.

**Figure 11:**
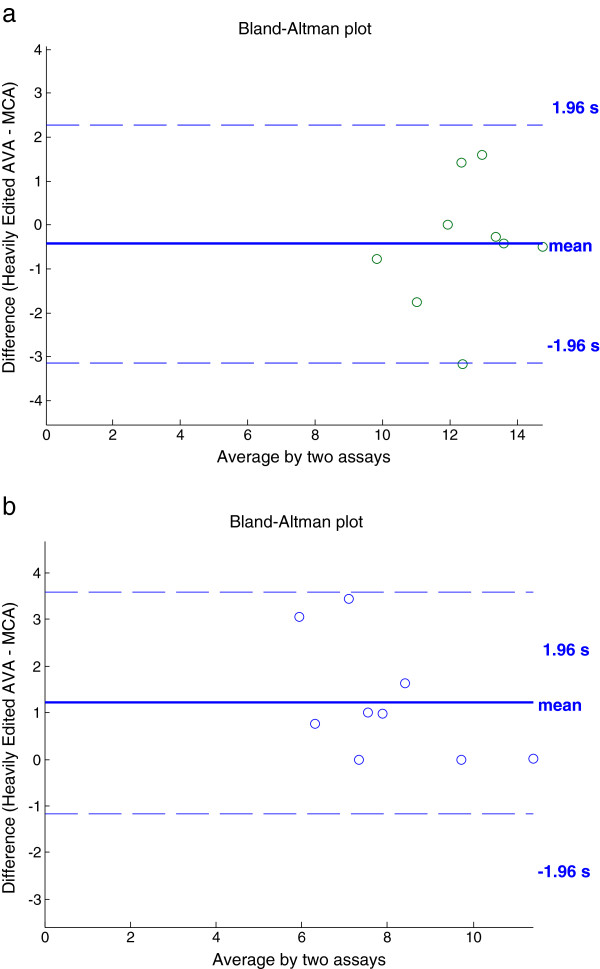
**Bland-Altman plots showing validity of fully-automated MCA as a measurement tool vs. current “gold standard” of heavily edited AVA (baseline (a) and hemorrhagic (b).a**: Bland-Altman plot showing correlation between heavily edited AVA and fully-automated MCA for baseline (PPV = 1) subjects. **b**: Bland-Altman plot showing correlation between heavily edited AVA and fully-automated MCA for hemorrhagic (PPV < 1) subjects.

To clearly understand the results generated by the proposed experimental algorithm (MCA) and heavily edited AVA, a chart is generated showing the FCD values for healthy baseline and hemorrhage conditions. Figure 7 shows the results from the heavily edited AVA software. The FCD values from subjects in the healthy baseline condition (PPV = 1) are labeled as baseline. Results of MCA are displayed in Figure 8. The decrease in FCD values for hemorrhage (PPV < 1) is clearly visible for each subject for the FCD values calculated by the experimental MCA automated algorithm. Figures 9 and 10 provide an example of the overlay between MCA and the edited semi-automated AVA method. However, comparing the compilation of data shown in Figures 7 and 8 and Tables 1 and 2, we conclude that MCA provides a better separation of FCD values between healthy baseline and hemorrhage. Furthermore, a Bland-Altman comparison of heavily edited AVA vs. fully-automated MCA (Figure 11) shows that MCA is capable of producing results in line with those achieved from edited AVA.

### Discussion

This study presents an entirely automated method, MCA, to derive quantitative information from microcirculatory videos in near real-time. Currently available techniques for analysis of the microcirculation, in their present state, do not appear to be practical in the clinical setting due to the need for significant manual interaction with the software in order to process the image and determine FCD. The significant difference in calculated FCD values across healthy baseline and hemorrhage shows that MCA has the potential for analyzing microcirculation videos in the clinical settings. Although the sample size of this study is relatively small, the algorithm demonstrates promise in its ability to rapidly provide quantitative information. Future studies will test the MCA algorithm on larger datasets—including human microcirculation videos—and improved accordingly. To overcome the variance among different video recordings, machine learning techniques, in particular neural networks and support vector machines that show superior performance in detection of elongated vessel-like objects in both biomedical image processing applications [32,33] as well as other image processing applications [34], will be applied and algorithm parameters adjusted accordingly. The use of machine learning techniques provide for a means to compensate for variations due to differences in factors such as lighting, pressure, video quality and specific machine/camera used for imaging. Notwithstanding the small sample size, the results show promise for an automated system that derives diagnostically important information from microcirculation videos.

MCA and semi-automated AVA both show a significant decrease in FCD values for the hemorrhagic subjects. Even though FCD values calculated using semi-automated AVA are statistically different for the healthy and hemorrhagic cases (μ=3.78, σ=2.267, t8=5.0, p-value = 1.1 × 10−3), the difference is not consistent (Figure 7). As noted in Figure 9, the overlay of analyzed results demonstrates very close performance between the automated MCA algorithm and that of the heavily edited AVA method. FCD values during hemorrhage generated by MCA were consistently lower than FCD values from the healthy baseline state (Figure 8). Visual inspection of the videos confirmed the ability of MCA to identify more vessels without flow and thus not include them in the determination of FCD. A potential limitation to this finding is new version of AVA that reportedly allows users to add missed vessels by manual drawing. We did not have access to this version. While use of this newer version may have reduced the differences between semi-automated AVA and the fully automated MCA, this improved version of AVA still requires editing and interaction of the user with the software.

While the approach taken with MCA cannot be considered actual real-time, the 20 second wait for results versus the 20-40 minutes of manual interaction required with semi-automated AVA makes the use of MCA near real-time and may thus be appropriate in the future for bedside point-of-care decision making. Additionally, the use of MCA would negate the considerable training that must be provided to the AVA user in order for them to be able to properly identify the active vessels in the results. This is in contrast to the proposed automated system, which incorporates that knowledge in the algorithm.

In the future, the automated method could be easily integrated into existing SDF or OPSI hardware systems which would allow real-time bedside determinations of FCD and potentially other microcirculatory parameters such as flow quantification. It is likely that in order to use SDF or OPSI derived FCD measures to affect care, an automated and reproducible software approach to analysis will be required for regulatory approval of such an approach.

Recent automated capillary detection methods such as Bezemer et al. [35] demonstrated impressive speed in its analysis. The authors of this method, however, indicate that performance is limited by high cell densities and velocities, which severely impede the applicability of this method in real SDF images. We believe this is due to the fact that many factors and thresholds in this method were set to fixed numbers and that they require adjustment from one video to another (just as in AVA). Again, these issues form the basis for the MCA approach as a means of reaching true automation. While we used a binary assessment of flow (flow or no-flow) in identifying functional capillaries, improvements in assessing flow beyond this simple method may be helpful. This might result in improved accuracy and compensation for some of the mechanical shortcomings of image acquisition using the camera technology, which, is capable of producing pressure related flow artifacts.

## Conclusions

A suggested algorithm to analyze microcirculation video recordings based on advanced machine learning is proposed which is capable of identifying active capillaries and calculating FCD parameters automatically. The approach is capable of detecting significant changes in FCD produced by hemorrhage and are comparable to a heavily manually edited commercially available software product. Future work will focus on adjusting the algorithm parameters on larger datasets and improving accuracy as well as developing improved methods of quantifying blood flow. It is hoped that these expanded methods and analyses will lead to the ability to derive diagnostically important decisions from the microcirculatory video recordings as well as to guide therapeutic interventions.

## Competing interests

Dr. Demir has intellectual property including pending patents on the technology discussed in this manuscript through Virginia Commonwealth University. She also has served as an employee of Signal Processing Technologies, LLC.

Dr. Hakimzadeh has intellectual property including pending patents on the technology discussed in this manuscript. She also is a co-owner/share-holder of Signal Processing Technologies, LLC which received an NSF SBIR grant for this research. She also serves as Chief Executive Officer at the company.

Dr. Hargraves has intellectual property including pending patents on the technology discussed in this manuscript. She also is a co-owner/share-holder of Signal Processing Technologies, LLC and serves as Chief Technology Officer at the company.

Dr. Ward has intellectual property including pending patents on the technology discussed in this manuscript through Virginia Commonwealth University. He also has serves as an uncompensated consulted to Signal Processing Technologies (SPT), LLC. Dr. Ward used to serve as Chief Medical Officer for SPT with ownership interest but currently he neither has any ownership in SPT nor serves as an officer for this company.

Mr. Myer has intellectual property including pending patents on the technology discussed in this manuscript through Virginia Commonwealth University. He has also served as an employee of Signal Processing Technologies, LLC.

Dr. Najarian has intellectual property including pending patents on the technology discussed in this manuscript through Virginia Commonwealth University. His wife is a co-owner/share-holder of Signal Processing Technologies, LLC, which received an NSF SBIR grant for this research. Dr. Najarian has also served as a technology advisor for Signal Processing Technology LLC.

## Authors’ contributions

Dr. SUD participated in the design of the image reprocessing algorithms, implemented the algorithms and processed the videos using the proposed algorithm. Dr.RH supervised the coordination of the entire study and the design and testing of the software. Dr. RHH co-supervised the design, implementation, testing and validation of the vessel detection algorithms and identification of pixels in vessels based on angle, intensity and width. Dr. KRW formed the guidelines for data collection, provided the data, outlined the clinical objectives of the study, and assessed the results from physiological and clinical standpoint and oversaw the general development and refinement of the image processing algorithm. Mr. EVM designed the improvements to the per-processing steps, participated in conducting the comparison between the proposed method and other methods, and carries out the statistical analysis. Dr. KN supervised the design of the entire image processing algorithm, in particular stabilization and region growing. All authors’ read and approved the final manuscript.

## Pre-publication history

The pre-publication history for this paper can be accessed here:

http://www.biomedcentral.com/1471-2342/12/37/prepub
